# Monitoring Disassembly and Cargo Release of Phase-Separated Peptide Coacervates with Native Mass Spectrometry

**DOI:** 10.1021/acs.analchem.3c02384

**Published:** 2023-07-13

**Authors:** Carmine P. Cerrato, Axel Leppert, Yue Sun, David P. Lane, Marie Arsenian-Henriksson, Ali Miserez, Michael Landreh

**Affiliations:** † Department of Microbiology, Tumor and Cell Biology, Karolinska Institutet − Biomedicum, Solnavägen 9, 17165 Solna, Sweden; ‡ Biological and Biomimetic Material Laboratory (BBML), Center for Sustainable Materials (SusMat), School of Materials Science and Engineering, Nanyang Technological University (NTU), Singapore, Singapore 637553; § School of Biological Sciences, Nanyang Technological University (NTU), Singapore, Singapore 637553; ∥ Department of Cell and Molecular Biology, Uppsala University, Box 596, 751 24 Uppsala, Sweden

## Abstract

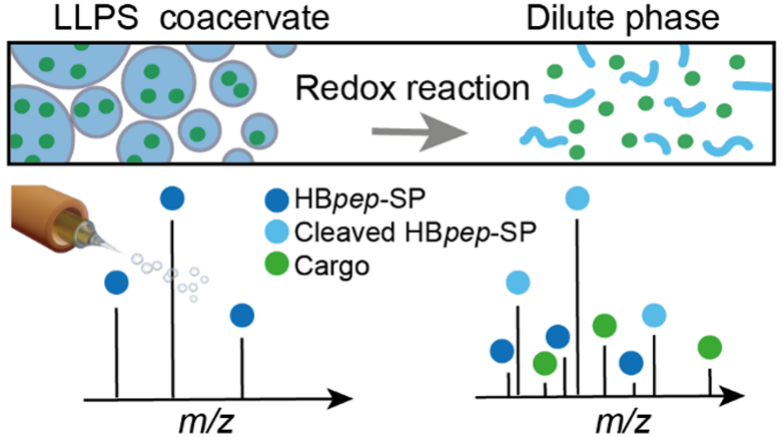

Engineering liquid–liquid phase separation (LLPS) of proteins and peptides holds great promise for the development of therapeutic carriers with intracellular delivery capability but requires accurate determination of their assembly properties *in vitro*, usually with fluorescently labeled cargo. Here, we use mass spectrometry (MS) to investigate redox-sensitive coacervate microdroplets (the dense phase formed during LLPS) assembled from a short His- and Tyr-rich peptide. We can monitor the enrichment of a reduced peptide in dilute phase as the microdroplets dissolve triggered by their redox-sensitive side chain, thus providing a quantitative readout for disassembly. Furthermore, MS can detect the release of a short peptide from coacervates under reducing conditions. In summary, with MS, we can monitor the disassembly and cargo release of engineered coacervates used as therapeutic carriers without the need for additional labels.

Liquid–liquid phase separation (LLPS), the assembly of partially disordered (bio)macromolecules into liquid-like condensates (also called coacervates), is a widespread phenomenon in biology. LLPS has been observed in processes ranging from the formation of membraneless cellular organelles to spinning of spider silk.^1−3^ Besides its importance in biology, LLPS is also becoming of growing interest for biotechnological purposes.^4−6^ One such application is the use phase-separating peptides to encapsule proteins or RNA and transport them across the cell membrane.^7^ Engineering peptides with specific properties that enable extracellular assembly and intracellular release thus represents a promising strategy for the delivery of a wide range of therapeutics. Histidine-rich squid beak-derived peptides (HB*peps*) exhibit fast and efficient phase separation into coacervates and concomitant recruitment of therapeutic cargo within the resulting microdroplets.^8,9^ We have recently developed an HB-derived peptide with a disulfide bond-containing self-immolative moiety that triggers disassembly of the droplets under reducing conditions found in the cell (Figure 1A).^10^ During assembly of the peptide, termed HB*pep*-SP, into coacervates under physiological conditions, therapeutic proteins, peptides, or nucleic acids are instantaneously recruited within the coacervates and are subsequently efficiently released upon entering the cytosol.

**Figure 1:**
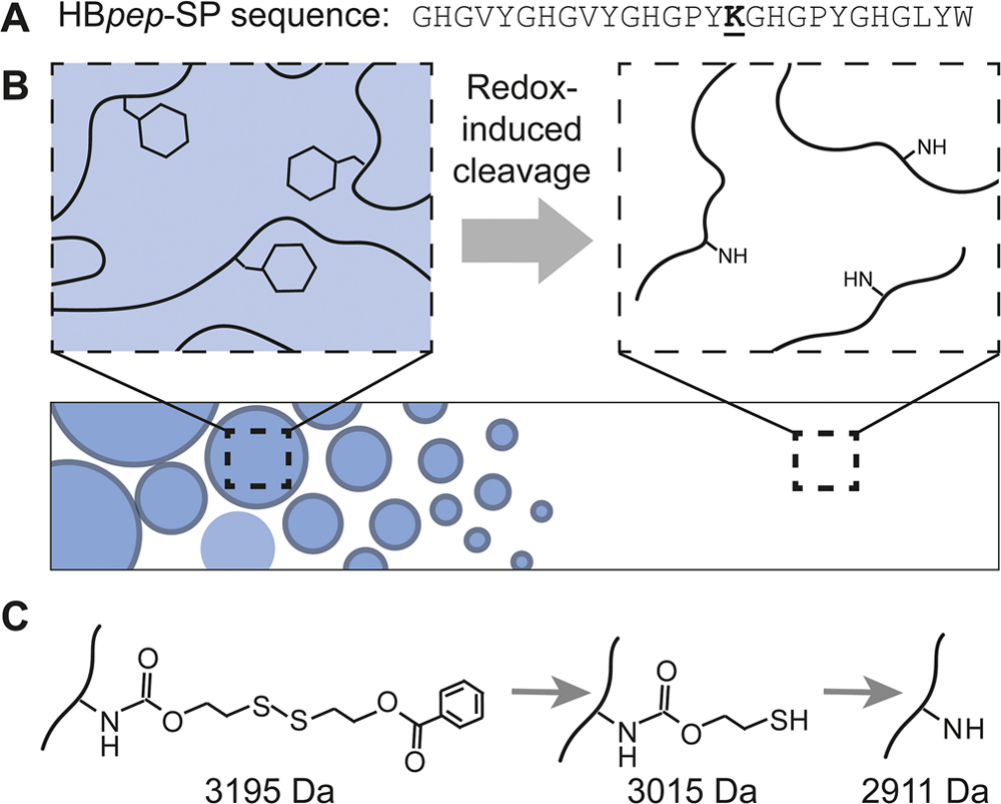
HB*pep*-SP drug delivery system. (A) Sequence of HB*pep*-SP. The lysine residue modified with a self-immolative moiety is underlined. (B) Principle of redox-controlled phase separation and disassembly of HB*pep*-SP coacervates. The self-immolative moiety enables coacervate formation by increasing the hydrophobicity of HB*pep*-SP. Disulfide reduction results in cleavage of the moiety and abolishes LLPS. (C) Chemical structures of the modified lysine residue, the disulfide-cleaved form, and the restored lysine amine group. The theoretical masses of each form are indicated.

Importantly, the development of coacervates with specific properties requires monitoring disassembly and cargo encapsulation, e.g., with light or fluorescence microscopy, limiting its application to labeled substrates. Mass spectrometry (MS) has recently been shown to reveal insights into the structures and dynamics of other phase-separating protein systems, including stress granule scaffolds and spider silk.^11−13^ In the case of HB*pep*-SP, disassembly is accompanied by a two-step mass shift, in which the self-immolative moiety is cleaved by disulfide reduction to reduce hydrophobicity, followed by restoration of the amine group of the connecting lysine residue (Figure 1B).^10^ We therefore reasoned that MS may be able to reveal details of the disassembly process, including its kinetics.

As the first step, we investigated the redox-driven disassembly of HB*pep*-SP coacervates by light microscopy and MS. Diluting the peptide from an acetic acid stock solution in 2 M ammonium acetate, pH 8, resulted in a cloudy solution due to droplet formation, which was confirmed by bright-field microscopy (Figure 2A). We then incubated the coacervates in the presence of 100 mM DTT and monitored droplet morphology. After 20 min, we observed a minor increase in droplet size due to fusion, while the total number of droplets decreased continuously until virtually no droplets could be detected after 60 min and the solution had become visibly clear (Figure 2A). In the absence of DTT, droplets continued to grow in size for 60 min due to continued fusion. To monitor the chemical conversion of HB*pep*-SP, aliquots were taken from the incubation in DTT at specific time points, diluted in methanol to dissolve any coacervates, and analyzed by nanoelectrospray ionization MS (nESI-MS, Figure 2B). We observed a clear mass shift from 3195 Da (HB*pep*-SP conjugated to the self-immolating moiety) to 3015 Da (cleavage by disulfide reduction). After 2.5 h, only the reduced form and a very minor population of the free lysine form could be detected (Figure 2B). These results indicate that in the present solvent system cleavage of the disulfide bond is sufficient to abolish LLPS and is largely independent of the introduction of an additional charge at the lysine side chain. These findings strongly indicated that we could use MS to directly quantify droplet disassembly. Plotting the percentage of reduced HB*pep*-SP at DTT concentrations of 10, 50, and 100 mM as a function of incubation time resulted in sigmoidal curves from which the half-times of the conversion (τ_1/2_) values of 76 min for 10 mM, 46 min for 50 mM, and 34 min for 100 mM DTT were obtained (Figure 2C, Figure S1).

**Figure 2:**
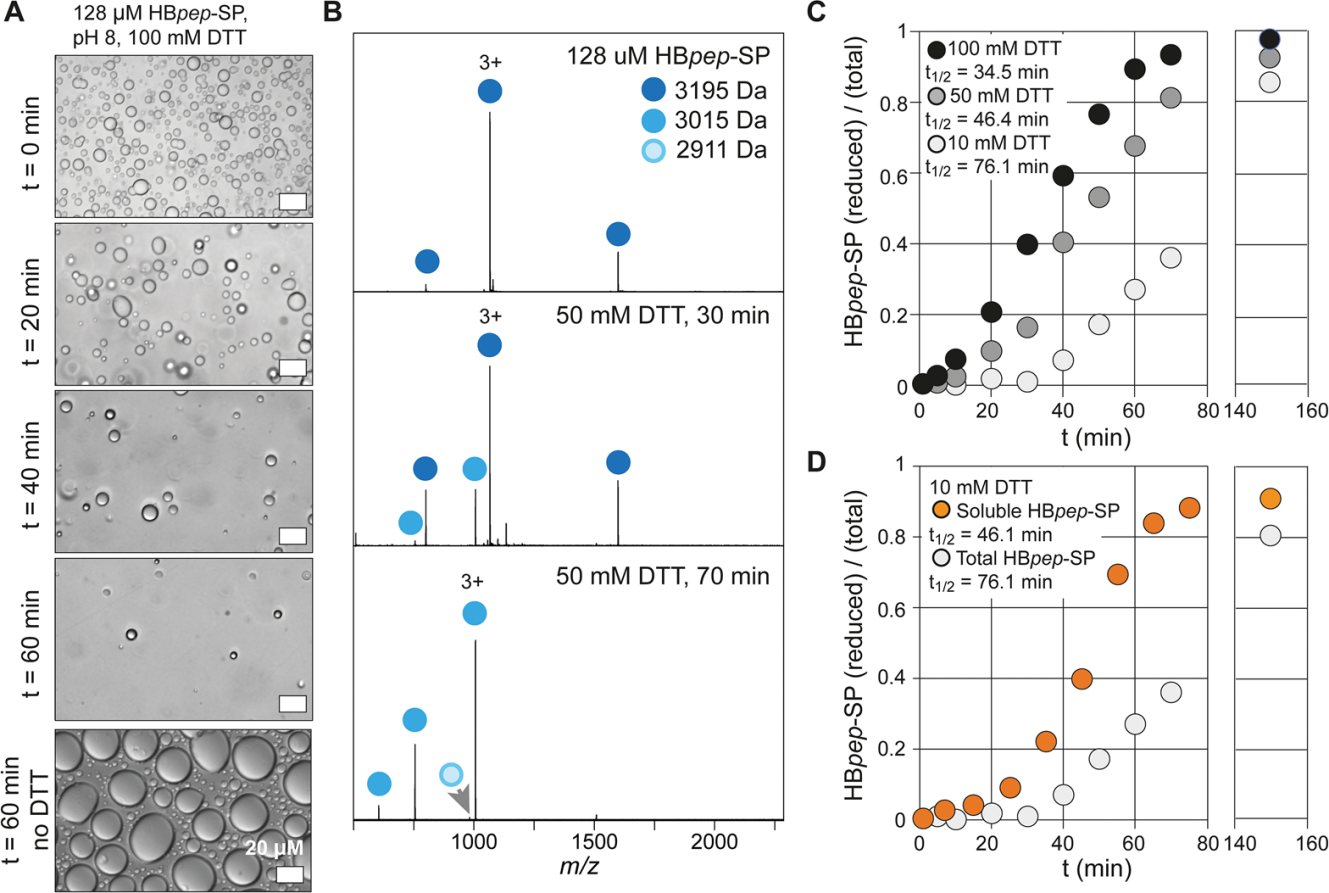
nanoESI-MS of HB*pep*-SP coacervates. (A) Light microscopy images of 128 μM HB*pep*-SP dissolved in 2 M ammonium acetate, pH 8, before reduction (top) and 20, 40, or 60 min after addition of 100 mM DTT show a reduction in the number of droplets over time. In the absence of DTT, droplets continue to grow due to fusion, and no dissolution is observed after 60 min. Scale bars are 20 μM. (B) Mass spectra of HB*pep*-SP coacervates dissolved in methanol before reduction (top) and 30 or 70 min after addition of 50 mM DTT (middle and bottom, respectively). A complete shift from the mass of the intact peptide (dark blue circles) to the mass of the disulfide-cleaved form (blue circles) occurs over 70 min, whereas only minor amounts of the restored lysine form (light clue circle) can be detected. (C) Quantification of HB*pep*-SP reduction over time for three different DTT concentrations. Data are plotted as the ratio of reduced peptide (3015 Da) to the total peptide (3195 and 3015 Da). (D) Native MS analysis of HB*pep*-SP coacervates in the presence of 10 mM DTT. Orange circles show the ratio of reduced to total (reduced + conjugated) peptide in the dilute phase. The ratio of reduced to total peptide is shown for the total protein population of the same solution, which was determined by denaturing MS (white circles, same as in Figure 3C). Comparison of the two curves reveals an accumulation of reduced peptide in the dilute phase.

To gain a more detailed picture of the disassembly process, we turned to native MS. Here, peptides are transferred from near-physiological solution conditions into the gas phase without distorting their noncovalent interactions.^14^ Importantly, peptides in the droplet state remain largely undetected under gentle ionization conditions, allowing us to instead monitor the soluble monomers that are in equilibrium with the droplet state.^12^ Taking advantage of the fact that ammonium acetate is MS compatible, we could therefore directly follow the release of reduced HB*pep*-SP during the DTT-induced coacervate disassembly. At 10 mM, the lowest DTT concentration tested, reduced peptide could be detected in the dilute phase after approximately 20 min. After 70 min, the dilute phase was found to consist of greater than 80% of reduced peptide, although it constituted less than 40% of the total peptide population in the sample (Figure 2D, Figure S1). From this finding, we conclude that the cleavage of the self-immolative moiety drives the release of the peptide from the coacervates. The enrichment of reduced HB*pep*-SP in the dilute phase and the concomitant reduction in the number of droplets suggest that disulfide cleavage changes the equilibrium between assembled and soluble form. Disulfide cleavage should occur rapidly in the dilute phase; however, the slow increase in reduced peptide suggests that its incorporation into coacervates slows down the reaction. The peptide likely exchanges between the soluble phase and the dense phase, and hence, reduction of the peptide in the dilute phase may shift the equilibrium toward the soluble form, leaving less and less conjugated peptide available for coacervate formation.

Having established that we can follow the disassembly process of HB*pep*-SP coacervates with native MS, we asked whether we could also detect cargo loading and release. As a test case, we employed a 51-residue disulfide-linked peptide from the proto-oncoprotein c-MYC, termed pepMYC (Figure 3A). For coassembly into coacervates, we mixed pepMYC with a 13-fold excess of HB*pep*-SP under denaturing conditions, after which we induced LLPS by adding a 20-fold excess of 2 M ammonium acetate, pH 8. Fluorescence microscopy of Cy5-labeled pepMYC confirmed incorporation of the peptide into the coacervates (Figure S2). We asked whether colocalization could be attributed to a direct interaction between both peptides or phase separation of pepMYC. However, AlphaFold2 predictions did not indicate complexes between dimeric pepMYC and HB*pep*-SP monomers, as evident from the low plDDT scores (Figure S2) and the fact that both peptides are positively charged at neutral pH, with a pI 9.7 and 8.4 for pepMYC and HB*pep*-SP, respectively. Furthermore, the FuzDrop server, which predicts phase-separating motifs, suggests that pepMYC has a very low propensity for LLPS. Native MS analysis of the HB*pep*-pepMYC coacervate solution showed strong signals for HB*pep*-SP, while pepMYC was virtually undetectable (Figure 3B), suggesting that little of the cargo peptide remained in the dilute phase. When pepMYC was instead added to HB*pep*-SP coacervates that had been preformed in ammonium acetate, we readily detected the intact pepMYC dimer in the dilute phase (Figure 3B). The data indicate efficient incorporation of pepMYC into coacervates during assembly, while the uptake into preformed coacervates by adsorption appears to be less effective. We next tested whether we could detect cargo release under denaturing and native conditions (Figure 3C). Mass spectra of the pepMYC-loaded HB*pep*-SP coacervates dissolved in 80% methanol showed peaks corresponding in mass to HB*pep*-SP as well as the intact pepMYC dimer, both with a slightly higher charge than that in ammonium acetate. Disassembly of the pepMYC-loaded HB*pep*-SP coacervates under native conditions was carried out by incubation in 50 mM DTT for 150 min, after which we detected both cleaved HB*pep*-SP and monomeric pepMYC by MS, indicating complete disulfide reduction of the carrier and cargo peptides. Unfortunately, the extreme excess of coacervate peptide over cargo peptide prevented reliable quantification of the release over time. However, MS captures encapsulation and release of pepMYC from HB*pep*-SP coacervates under nondenaturing solution conditions.

**Figure 3:**
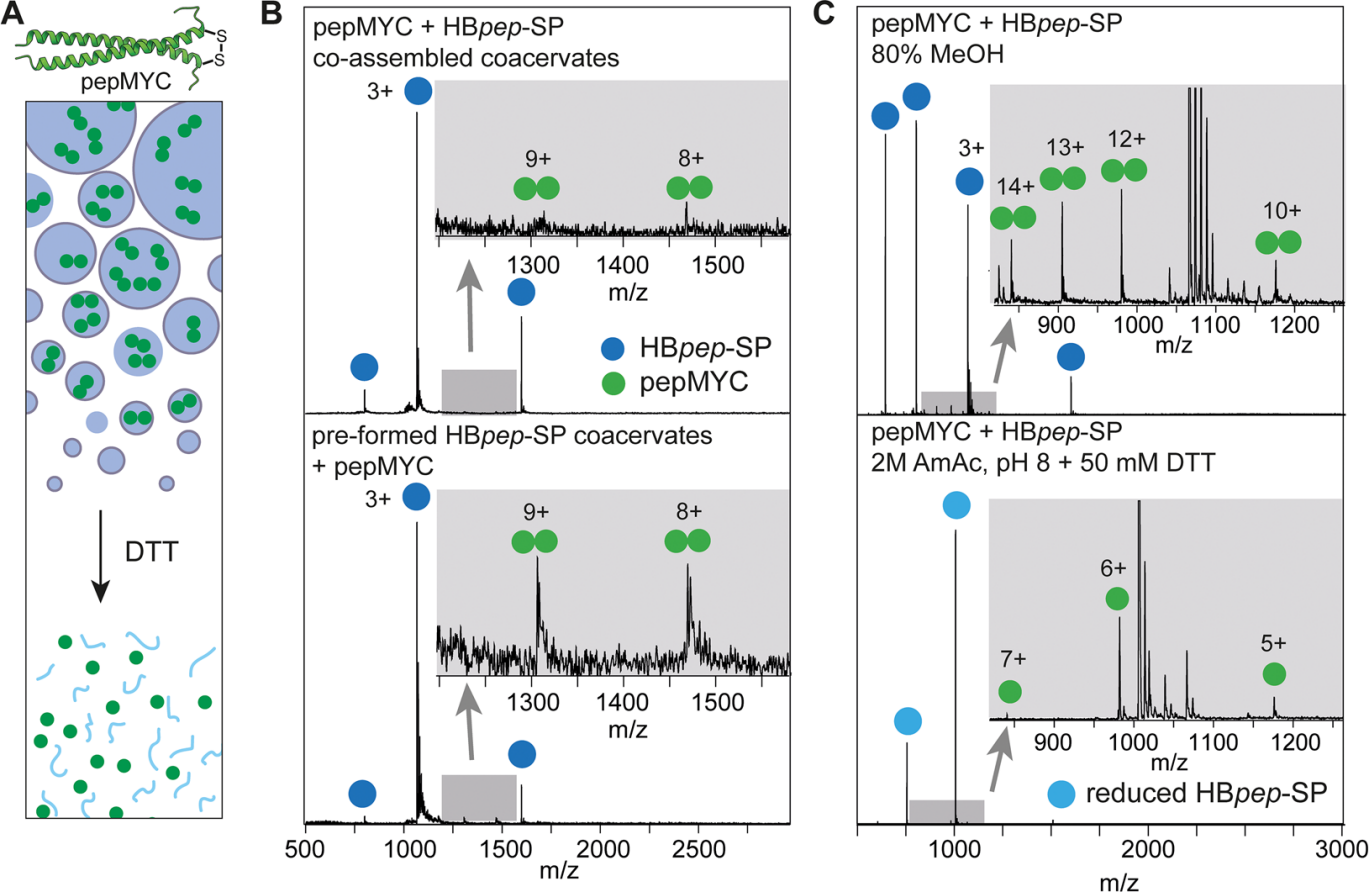
Cargo loading and release from HB*pep*-SP coacervates. (A) Top: AlphaFold2 model of the pepMYC dimer with the disulfide bond indicated. Bottom: Principle of coassembly and release of pepMYC (green) and HB*pep*-SP (blue) coacervates. (B) Top: Native MS of coacervates of pepMYC and HB*pep*-SP coassembled under native conditions shows only minor peaks for the pepMYC dimer (gray magnification). Bottom: Native MS of pepMYC added to preassembled HB*pep*-SP coacervates recorded under the same instrumental conditions shows clear peaks corresponding in mass to pepMYC. (C) Top: Mass spectra of pepMYC-loaded HB*pep*-SP coacervates dissolved in 80% methanol show the presence of highly charged pepMYC dimers. Bottom: native mass spectra of pepMYC-loaded HB*pep*-SP coacervates following 150 min incubation in 50 mM DTT indicate the release of disulfide-cleaved, monomeric pepMYC (green circles) and HB*pep*-SP (light blue circles) into the dilute phase.

## Conclusions

Engineering phase separating systems for intracellular drug delivery requires a detailed understanding of their assembly, cargo loading, and release. Here, we demonstrate that MS can capture the redox-driven disassembly of coacervates and detect loading and release of a drug-like cargo peptide. By combining native and denaturing MS, we detect changes in the peptide populations of the condensed and the dilute phases, which can be employed to develop peptides with specific assembly properties and tunable release kinetics of cargos. Importantly, the approach is label-free, scalable, and in principle independent of the instrument platform. We believe that MS can open new avenues for understanding LLPS and its applications in biotechnology and therapeutic development.

## Supporting Information Available

Experimental procedures, fitting data for all curves in Figure 2C and D, fluorescence microscopy of HB*pep*-SP and pepMYC coacervates, and AlphaFold and FuzDrop prediction data (PDF)

## Abbreviations

LLPS: liquid–liquid phase separation
MS: mass spectrometry
DTT: dithiothreitol
nESI: nanoelectrospray ionization
