# Supplementary material for: Monitoring Disassembly and Cargo Release of Phase-Separated Peptide Coacervates with Native Mass Spectrometry
Source: Anal Chem. 2023 Jul 13;95(29):10869–72. doi: 10.1021/acs.analchem.3c02384 (PMC10372869; doi:10.1021/acs.analchem.3c02384)
Supplement: Supplementary file 1 — ac3c02384_si_001.pdf [file ac3c02384_si_001.pdf]

Supporting information

# Monitoring disassembly and cargo release of phase-separated peptide coacervates with native mass spectrometry

Carmine P. Cerrato[1], Axel Leppert[1], Yue Sun[2], David P. Lane[1], Marie Arsenian-Henriksson[1], Ali Miserez[2,3,*], and Michael Landreh[1,4,*]

[1] Department of Microbiology, Tumor and Cell Biology, Karolinska Institutet – Biomedicum, Solnavägen 9, 17165 Solna, Sweden

[2] Biological and Biomimetic Material Laboratory (BBML), Center for Sustainable Materials (SusMat), School of Materials Science and Engineering, Nanyang Technological University (NTU), Singapore, Singapore

[3] School of Biological Sciences, NTU, Singapore, Singapore.

[4] Department of Cell and Molecular Biology, Uppsala University, Box 596, 751 24, Uppsala, Sweden

* e-mail: ali.miserez@ntu.edu.sg or michael.landreh@icm.uu.se

**Table of Content**

| | |
|---|---|
| Experimental | Page S1 |
| Supporting References | Page S3 |
| Supporting Figure 1 | Page S4 |
| Supporting Figure 2 | Page S5 |

## Experimental

*Peptides.* HB*pep*-SP was produced as described [1]. Amino-terminally acetylated and carboxy-terminally amidated pepMYC (sequence: Ac-KAPKVVILKKATAYILSVQAETQKLISEIDLLRKQNE QLKHKLEQLRNSC(Cy5)A-$NH_2$) with and without Cys-linked Cy5 label was purchased from Pepscan Presto B.V. (The Netherlands) and dissolved in $dH_2O$ to a final concentration of 100 μM. HB*pep*-SP coacervates were prepared by dissolving 0.5 mg lyophilized peptide in 50 μL acetic acid (Sigma). To initiate coacervate assembly, 2 μL HB*pep*-SP stock solution were diluted in 48 μL 2M ammonium acetate (Sigma) adjusted to pH 8 with 25% ammonia solution (Sigma). DTT was added from a 2M stock solution in $dH_2O$ to final concentrations of 10, 50, or 100 mM. HB*pep*-SP coacervates with pepMYC were assembled by mixing 2 μL HB*pep*-SP stock solution in acetic acid with 8 μL pepMYC stock solution (100 μM) in $dH_2O$, followed by the addition of 40 μL 2M ammonium acetate, pH 8.

*Bright-field microscopy.* For brightfield microscopy, a solution 128 μM HB*pep*-SP in 2M ammonium acetate, pH 8, and 100 mM DTT was prepared. Samples were resuspended thoroughly (10x pipetting up and down) every 10 min. At the corresponding time point, 50 μL were transferred into one well of a 96-well half-area low-binding plate (Corning) with transparent bottom. The samples were allowed to sediment for 1 min and images were taken. images were acquired using a Nikon Eclipse Ti series confocal microscope (Nikon Melville, NY, USA) equipped with an S Plan Fluor ELWD 40X DIC N1/0.6 NA (Nikon Melville, NY, USA), and a Zyla sCMOS camera (Andor, Oxford Instruments, Belfast, UK).

*Fluorescence microscopy.* Fluoro-labelled pepMYC (pepMYC-Cy5, sequence in Table Sx.) with $\lambda_{ex}$ 647 nm; $\lambda_{em}$ 665 nm (laser $\lambda_{ex}$ 640) was used to visualise the incorporation of peptide in coacervates. Samples were prepared as described for the coacervates formation with unlabelled peptide. Images were acquired with a Nikon Eclipse Ti series confocal microscope (Nikon Melville, NY, USA) equipped with a Plan Fluor 10x/0.3NA DIC L N1 and an S Plan Fluor ELWD 40X DIC N1/0.6 NA (Nikon Melville, NY, USA), a Full Multiband Quad filter (FF01-440/5221/607/700, Em) and a Zyla sCMOS camera (Andor, Oxford Instruments, Belfast, UK). Images were analysed using the NIS-Elements Advanced Research 5.02.03 software (Nikon, Melville, NY, USA). A minimum of 5 fields were captured for for each condition.

*Sample preparation for mass spectrometry.* For denaturing MS, 2 μL of the HB*pep*-SP coacervate solution were removed after 1, 2, 5, 10, 20, 30, 40, 50, 60, 70, and 150 min after addition of 10, 50, or 100 mM DTT, diluted in 3 μL methanol (Sigma), and directly injected into

the mass spectrometer. For native MS, 3 μL of the HB*pep*-SP coacervate solution were removed after 1, 3, 7, 15, 25, 35, 45, 55, 65, 75, and 150 min after addition of 10 mM DTT. Alternating time-points for native and denaturing MS analysis were chosen to facilitate monitoring of soluble and total HB*pep*-SP from the same incubation.

*Mass spectrometry.* Mass spectra were acquired on a Micromass LCT ToF modified for analysis of intact protein complexes (MS Vision, The Netherlands) equipped with an offline nanospray source. ESI capillaries were purchased from Thermo. The capillary voltage was 1.5 kV, the cone voltage 50 V, and the RF lens 1.5 kV. The pressure in the ion source was maintained at 9.0 mbar. Spectra were visualized using MassLynx 4.1 (Waters, UK). Data were plotted using Microsoft Excel 16.58 with the Excel Solver tool [2]. HB*pep*-SP kinetics were fitted to an empirical sigmoidal function using the excel solver tool minimizing the SSR.

$$F = F_0 + A/(1 + \exp(r_{max}(\tau_{1/2} - t))) \quad (1)$$

where $F_0$ is the baseline value, A the amplitude, $r_{max}$ the maximum growth rate, and $\tau_{1/2}$ the peptide conversion half time.

## Supporting References


(1) Sun, Y.; Lau, S. Y.; Lim, Z. W.; Chang, S. C.; Ghadessy, F.; Partridge, A.; Miserez, A. Phase-Separating Peptides for Direct Cytosolic Delivery and Redox-Activated Release of Macromolecular Therapeutics. *Nat. Chem.* **2022**, *14*, 274–283.

(2) Kemmer, G.; Keller, S. Nonlinear Least-Squares Data Fitting in Excel Spreadsheets. *Nat. Protoc.* **2010**, *5*, 267–281

(3) Mirdita, M.; Schütze, K.; Moriwaki, Y.; Heo, L.; Ovchinnikov, S.; Steinegger, M. ColabFold: Making Protein Folding Accessible to All. *Nat. Methods* **2022**, *19*, 679–682.

(4) Hardenberg, M.; Horvath, A.; Ambrus, V.; Fuxreiter, M.; Vendruscolo, M. Widespread Occurrence of the Droplet State of Proteins in the Human Proteome. *Proc. Natl. Acad. Sci. U. S. A.* **2021**, *117*, 33254–33262.

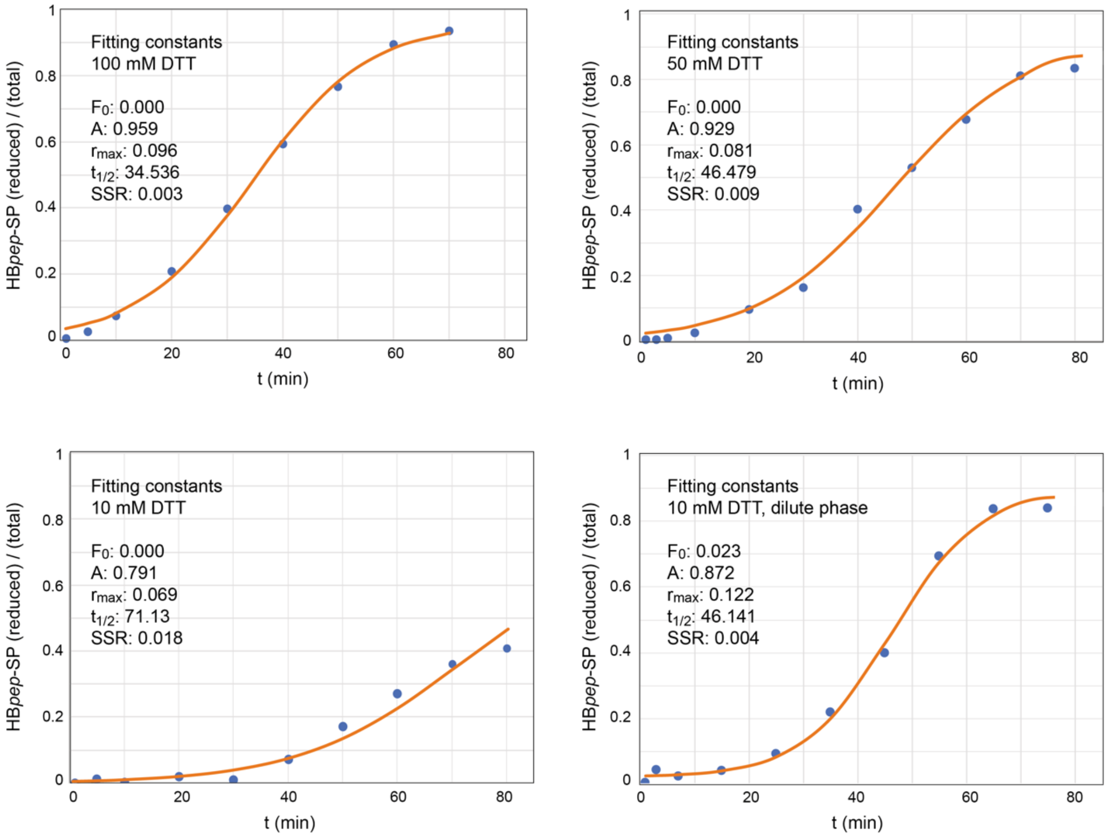


**Supporting Figure 1. Curve fits for the release of reduced HB*pep*-SP shown in Figure 2 C and D.** Fits were generated using Excel Solver. The fitting parameters ($F_0$ and A) and results ($r_{max}$, $\tau_{1/2}$ and square sum R) are shown on each graph.

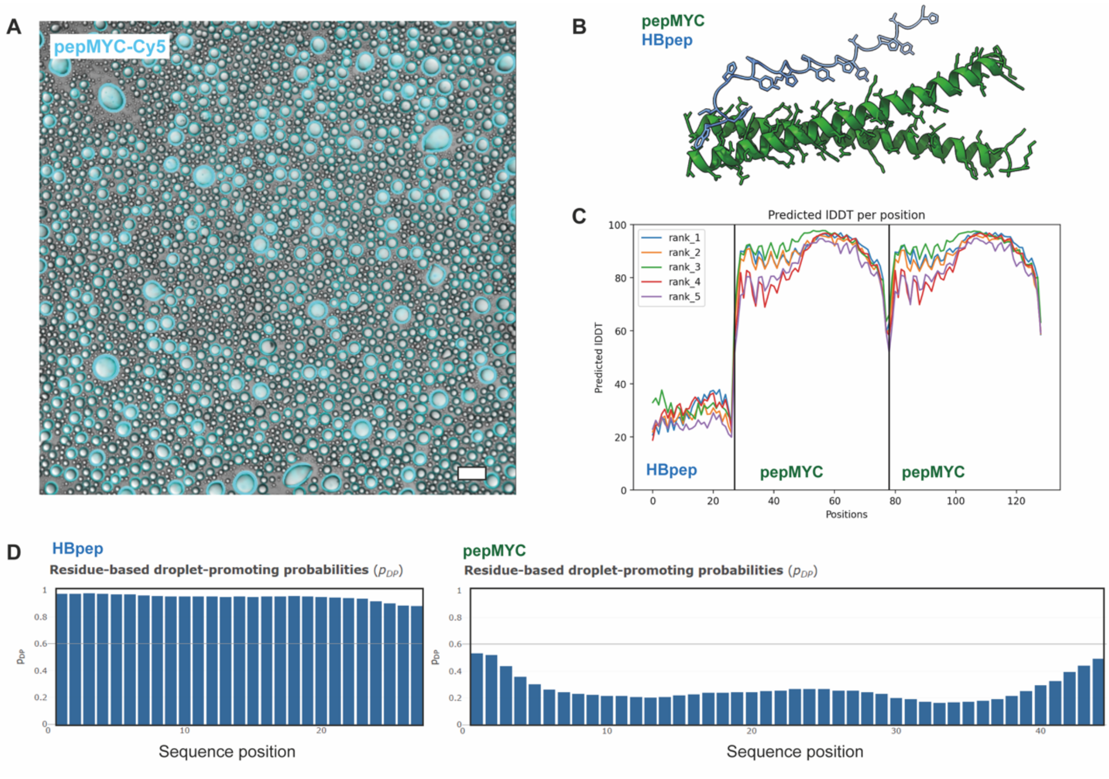


**Supporting Figure 2. Interactions of pepMYC with HB*pep*-SP.** (A) Overlayed bright-field and fluorescence microscopy image of coacervates formed with Cy5-labeled pepMYC (cyan) and incubated for 60 min show the presence of labeled cargo peptide in the HB*pep*-SP coacervates. Scale bar is 50 μm. (B) and (C) AlphaFold2 predictions [3] yield a high-confidence model for the pepMYC dimer, but no well-defined interactions with HB*pep*-SP. The top-scoring model and the piDDT scores are shown. (D) FuzDrop [4] scores for HB*pep*-SP (left) and pepMYC (right) indicate high LLPS propensity for the carrier, but no phase separation by the cargo peptide.
